# Cardiac and vascular effects of low-dose steroids during the early phase of septic shock: An echocardiographic study

**DOI:** 10.3389/fcvm.2022.948231

**Published:** 2022-09-26

**Authors:** François Bagate, Alexandre Coppens, Paul Masi, Nicolas de Prost, Guillaume Carteaux, Keyvan Razazi, Armand Mekontso Dessap

**Affiliations:** ^1^AP-HP, CHU Henri Mondor, DHU A-TVB, Service de Médecine Intensive Réanimation, Créteil, France; ^2^Université Paris Est Créteil, Faculté de Médecine, Groupe de Recherche Clinique CARMAS, Créteil, France; ^3^INSERM U955, Institut Mondor de Recherche Biomédicale, Créteil, France

**Keywords:** low-dose steroid therapy, hydrocortisone, fludrocortisone, septic shock, echocardiography, global longitudinal strain, ventriculo-arterial coupling

## Abstract

**Background:**

Low-dose steroids are known to increase arterial pressure during septic shock through restoration of vasopressor response to norepinephrine. However, their effects on cardiac performance and ventriculo-arterial coupling (VAC) have never been scrutinized during human septic shock. The aim of this study was to perform a comprehensive description of the cardiovascular effects of low-dose steroids using modern echocardiographic tools (including speckle tracking imaging).

**Methods:**

This prospective study was conducted in the intensive care unit (ICU) of a university hospital in France. Consecutive adult patients admitted for septic shock and requiring low-dose steroid therapy were prospectively enrolled within 24 h of septic shock onset. We recorded hemodynamic and echocardiographic data to explore left ventricle (LV) contractility, loading conditions and VAC just before the initiation of low-dose steroids (50 mg intravenous hydrocortisone plus 50 μg enteral fludrocortisone) and 2–4 h after.

**Results:**

Fifty patients [65 (55–73) years; 33 men] were enrolled. Arterial pressure, heart rate, almost all LV afterload parameters, and most cardiac contractility parameters significantly improved after steroids. VAC improved with steroid therapy and less patients had uncoupled VAC (> 1.36) after (24%) than before (44%) treatment.

**Conclusion:**

In this comprehensive echocardiographic study, we confirmed an improvement of LV afterload after initiation of low-dose steroids. We also observed an increase in LV contractility with improved cardiovascular efficiency (less uncoupling with decreased VAC).

## Introduction

Relative adrenal insufficiency, also appraised as critical illness-related corticosteroid insufficiency is common during septic shock. It is the main pathophysiological rationale for the use of low-dose corticosteroid therapy in septic shock (1). The anti-inflammatory (2) and vasoactive (3) properties of corticosteroids have been well known for several decades. Several studies have demonstrated the peripheral vascular effects of low-dose steroids in septic shock, including an increase in mean arterial pressure (MAP) through restoration of systemic vascular resistance and enhanced vasopressor response to norepinephrine (4–8).

In addition to vasoplegia, septic shock is also characterized by myocardial dysfunction, with a major interaction between contractility and loading conditions (9). Animal models of sepsis suggest a protective role for low-dose hydrocortisone in myocardial injury (10). However, the effect of low-dose steroids on cardiac performance has never been scrutinized during human septic shock. We hypothesized that low-dose steroid therapy may enhance cardiac contractility in addition to its vasopressive effects during human septic shock. Given the strong interaction between left ventricle (LV) contractility and loading conditions during human septic shock (9, 11), it is crucial to assess all components of cardiac performance in this setting. The aim of the present study was to perform a comprehensive description of the effect of low-dose steroid therapy on cardiac and vascular function (contractility, preload, and afterload, with their interactions) during human septic shock, using modern echocardiographic tools.

## Materials and methods

### Patients

We conducted a prospective observational monocenter study in the medical intensive care unit (ICU) of Henri Mondor University Hospital, Créteil, France, between August 8, 2018 and May 29, 2021. All consecutive patients admitted during this period for septic shock (as defined according to the American College of Chest Physicians (ACCP)/Society of Critical Care Medicine (SCCM) Consensus Conference (12) and requiring low-dose steroid therapy, according to guidelines (13, 14), were prospectively enrolled within 24 h of septic shock onset. Non-inclusion criteria were age less than 18 years, pregnancy, patients under protection (guardianship, curators or safeguard of justice), chronic heart failure with reduced left ventricular ejection fraction (LVEF < 45%) documented in patient’s history, severe valvular disease and prior corticosteroid therapy.

The study was approved on July 12, 2018 by the institutional ethics committee, Institutional Review Board (IRB) Mondor (reference number: 2201948) as a component of standard care, and patient consent was waived, as per French law. Written and oral information about the study was given to the families.

### Treatment protocol

Norepinephrine was the first-choice vasopressor therapy (used to target a MAP of 65 mmHg or more); dobutamine was added, based on physician decision, in the presence of decreased LVEF (<45%) with ongoing signs of hypoperfusion despite adequate MAP and correction of hypovolemia with absence of fluid responsiveness. Catecholamine dose and fluid management were kept constant between the two ultrasound assessments, unless significant hemodynamic instability; patients who required a change in norepinephrine dose and/or fluid loading during the study protocol were secondarily excluded. The steroid replacement therapy consisted of hydrocortisone 50 mg intravenously every 6 h plus enteral fludrocortisone (50 μg/day). The patients were followed up until ICU discharge.

### Echocardiography

Transthoracic echocardiography (TTE) was performed in early phase of septic shock after hemodynamic stabilization by trained operators (competent in advanced critical care echocardiography) (15). The TTE studies were performed on a GE Vivid S7 or E9 ultrasound system (GEMS, Buc, France), with a 1.5–4.5 MHz (M5S-D) transducer. The first TTE was performed at the initiation of steroid replacement therapy (just before first intravenous bolus of hydrocortisone 50 mg plus enteral fludrocortisone 50 μg). The second TTE was performed 2–4 h after the initiation of low-dose steroid therapy. The echocardiographic results were reported based on the Preferred Reporting Items for Critical care Echocardiography Studies (PRICES) statement of the European Society of Intensive Care Medicine (16). All individual measurements were averaged over a minimum of three cardiac cycles (five to ten in case of non-sinus rhythm) and collected at end-expiration.

### Assessment of contractility and loading conditions

#### Preload

Preload was assessed using estimates of LV filling pressures [*E*/*A* and *E*/*e’* ratios from pulsed-wave Doppler early (*E*) and late (*A*) and tissue Doppler early (*e’*) diastolic wave velocity at the lateral and septal mitral valve annulus] (17) and respiratory variations of velocity time integral (VTI) of the left ventricle outflow tract (LVOT) as a surrogate of fluid responsiveness (18, 19).

#### Afterload

Afterload was assessed using the following indices: (i) diastolic arterial pressure (DAP) which is often used as a surrogate of LV afterload in clinical practice (20); (ii) systemic vascular resistance (the most commonly used measure of vascular tone) (21) = $\frac{80*m\,e\,a\,n\,a\,r\,t\,e\,r\,i\,a\,l\,p\,r\,e\,s\,s\,u\,r\,e\,\left(m\,m\,H\,g\right)}{cardiacoutput\left(L.min^{-1}\right)}$; (iii) end-systolic arterial elastance (E_*a*_), to reflect the pulsatile component of peripheral load (22) = $\frac{0.9*s\,y\,s\,t\,o\,l\,i\,c\,a\,r\,t\,e\,r\,i\,a\,l\,p\,r\,e\,s\,s\,u\,r\,e\,\left(m\,m\,H\,g\right)}{s\,t\,r\,o\,k\,e\,v\,o\,l\,u\,m\,e\,\left(m\,L\right)}$; (iv) diastolic shock index (DSI), that could reflect the severity of circulatory dysfunction during vasodilatory conditions = $\frac{h\,e\,a\,r\,t\,r\,a\,t\,e\,\left(b\,p\,m\right)}{d\,i\,a\,s\,t\,o\,l\,i\,c\,a\,r\,t\,e\,r\,i\,a\,l\,p\,r\,e\,s\,s\,u\,r\,e\,\left(m\,m\,H\,g\right)}$) (23); and (v) LV end-systolic wall stress (to reflect the combined effects of peripheral loading conditions and factors internal to the heart according to Laplace’s principle) = $\frac{S\,y\,s\,t\,o\,l\,i\,c\,a\,r\,t\,e\,r\,i\,a\,l\,p\,r\,e\,s\,s\,u\,r\,e\,\left(m\,m\,H\,g\right).l\,e\,f\,t\,v\,e\,n\,t\,r\,i\,c\,l\,e\,e\,n\,d-s\,y\,s\,t\,o\,l\,i\,c\,v\,o\,l\,u\,m\,e\,\left(m\,L\right)}{1000}$ (21).

#### Systolic function and left ventricular contractility

LV systolic function was assessed using indices obtained from the following techniques: (i) two-dimensional echocardiography, with LVEF (computed from LV volume using bi-plane Simpson method (24) when image quality was good, or visually estimated when poor image quality did not allow sufficient identification of the endocardium) (25); (ii) tissue Doppler imaging, with peak systolic wave (s’) at the lateral and septal mitral valve annulus (26); (iii) speckle tracking imaging, with LV global longitudinal peak systolic strain (LV-GLS), derived from apical long-axis (three-, four,- and two-chamber) clips obtained with a frame rate ≥ 50 Hz whenever possible with on-line analyses conducted by two trained operators on the semi-automated EchoPAC package (GEMS, Buc, France); (iv) the LV end-systolic maximal elastance estimated by the modified single-beat method [E_*es(sb)*_], as proposed by Chen et al. (27).

#### Description of left ventricle end-systolic maximal elastance using single-beat method

The LV end-systolic maximal elastance (E_*es*_) is a major determinant of the LV systolic performance and the interaction between heart and vascular system. E_*es*_ corresponds to the slope of the end-systolic pressure-volume relation, which is classically invasively determined by catheter with LV pressures and volumes recorded under different cardiac loading conditions. Several alternatives have been proposed for estimating E_*es*_ without loading interventions, and these are generally referred to as single-beat methods [E_*es(sb*)_]. The algorithm for E_*es(sb)*_ proposed by Chen et al. (27) is based on the following steps and principles:

(a) If we consider LV elastances during isovolumic contraction [E_*d*_ = P_*d*_/(V_*d*_-V_0_)] and at end systole [E_*es(sb)*_ = P_*es*_/(V_*es*_-V_0_)], their ratio [E_*Nd*_ = E_*d*_/E_*es(sb)*_], called the time and amplitude normalized time varying elastance, has been shown to be conserved during the isovolumic contraction period in humans (28).

(b) If we consider the onset of ejection (end of isovolumic contraction) for E_*d*_, then E_*es(sb)*_ can be computed from (a) using E_*Nd*_, stroke volume (which equals V_*d*_-V_*es*_), diastolic arterial pressure (which equals P_*d*_) and systolic arterial pressure (which equals P_*es*_/0.9), as follows: E_*es(sb)*_ = [P_*d*_-(E_*Nd*_ × P_*s*_ × 0.9)]/[SV × E_*Nd*_] (27).

(c) To estimate E_*Nd*_, a regression model has been developed using systolic function (LVEF) and arterial load (ratio of arterial diastolic to systolic pressure), along with pre-ejection and ejection times, as follows: non-invasive estimated normalized left ventricular elastance at the onset of ejection [E_*Nd(est*)_] = 0.0275–0.165 × LVEF + 0.3656 × (P_*d*_/P_*s*_) + 0.515 × E_*Nd(avg)*_; where E_*Nd(avg*)_ (group-average normalized left ventricular elastance at the onset of ejection) = Σa_*i*_ × t_*Nd*_*^i;^* a_*i*_ values are 0.35695, –7.2266, 74.249, –307.39, 684.54. –856.92, 571.95, and –159.1 for *i* = 0 to *i* = 7, respectively; the t_*Nd*_ value is determined by the ratio of pre-ejection time (R-wave to flow onset) to total ejection time (R-wave to end-flow), with the time at onset defined by the aortic Doppler waveform (29).

#### Global function

We also assessed the heart-arterial interaction with the ventricular-arterial coupling (VAC) = $\frac{E\,a}{E\,e\,s\,\left(s\,b\right)}$ (30), as suggested by recent guidelines (31); a VAC > 1.36 was considered as reflecting uncoupling with an alteration of LV ejection efficiency (32, 33). LV stroke volume, cardiac output and cardiac index were derived from LVOT diameter and VTI. We also assessed cardiac power index (CPI), a parameter of global cardiac function representing the cardiac pumping ability which is an important determinant of outcome in cardiogenic shock = $\frac{meanarterialpressure{\left(mmHg\right)}^{*}cardiacindex\left(L.min^{-1}.m^{-2}\right)}{145}$ (34, 35). Last, an afterload-adjusted LV ejection fraction (LVEF_*EA*_) was assessed using a simple non-linear approach as recently proposed = $L\,V\,e\,j\,e\,c\,t\,i\,o\,n\,f\,r\,a\,c\,t\,i\,o\,n^{*}\,\,\sqrt{\text{arterial}}\,\mathrm{elastance}$ (36).

### Other variables collected

The following data were collected at inclusion: age, sex, body mass index, past medical history, standard treatments, Sequential Organ Failure Assessment (SOFA) score (37), Simplified Acute Physiologic Score (SAPS) II (38). In addition, we collected septic shock characteristics, hemodynamic parameters (blood pressure and heart rate before and after steroid administration, fluid balance, dose of norepinephrine, inotrope need, arterial blood lactate, cardiac rhythm, ventilatory support with respiratory mechanics (mode of ventilation, tidal volume, respiratory rate, positive end expiratory pressure, plateau pressure, driving pressure, respiratory system compliance) for patients under invasive ventilation at the time of the echo examinations. Moreover, the following outcomes were recorded: renal replacement therapy, presence of acute respiratory distress syndrome (ARDS) as per the Berlin definition and vital status at ICU discharge.

### Definitions

We defined septic cardiomyopathy as the appearance at echocardiography of hypokinesia (LVEF < 45% or when inotrope infusion was needed to achieve a value ≥ 45%) during the first day of septic shock with the absence of acute coronary syndrome or history of chronic heart failure in the medical charts. We assessed the change in MAP between baseline and 2–4 h after steroid administration. We defined steroid MAP responders (non-responders) as patients with a relative increase in MAP above (below) the median value of the cohort.

### Statistical analysis

Statistical analyses were performed with the JMP software (version 14; SAS Institute Inc., Cary, NC) and Graph-Pad Prism software (version 8; GraphPad Software Inc., La Jolla, CA, USA). The primary endpoint of this study was the change in LV contractility after the first administration of low-dose steroid therapy. For the analysis of the primary endpoint, the LV-GLS was chosen for its robustness, reproducibility and sensitivity. We calculated that a sample size of at least 43 patients would have a 90% power to detect a 20% improvement in LV-GLS after low-dose steroid therapy initiation, considering a baseline LV-GLS of –14% with a standard deviation (SD) of 4%, based on previous echocardiographic studies in septic shock patients (39–41). Taking into account the feasibility of LV-GLS in our ICU (42), we therefore planned to include a total of 50 patients. In addition, we performed subgroup analyses. We compared the evolution of echocardiographic and hemodynamic parameters in response to low-dose steroids according to the presence on septic cardiomyopathy and according to MAP response. Descriptive statistics are presented as mean (+/–SD), median (interquartile range), or proportion, based on data type and distribution. Normality of continuous variables was assessed with the Shapiro–Wilk test. Comparisons of categorical variables were made using the Chi-2 test or Fisher exact test, as appropriate. Continuous variables were compared using a *T*-test or Mann-Withney test for independent variables, and a paired *T*-test or paired Wilcoxon test for paired variables, with Benjamini-Hochberg correction for multiple comparisons. The reproducibility of some echocardiographic variables in our laboratory was assessed using the British Standards Institution coefficient (twice the standard deviation of the differences in repeated measurements) (43). A *p*-value < 0.05 in bilateral configuration was considered for statistical significance.

## Results

### Patient characteristics

**FIGURE 1:**
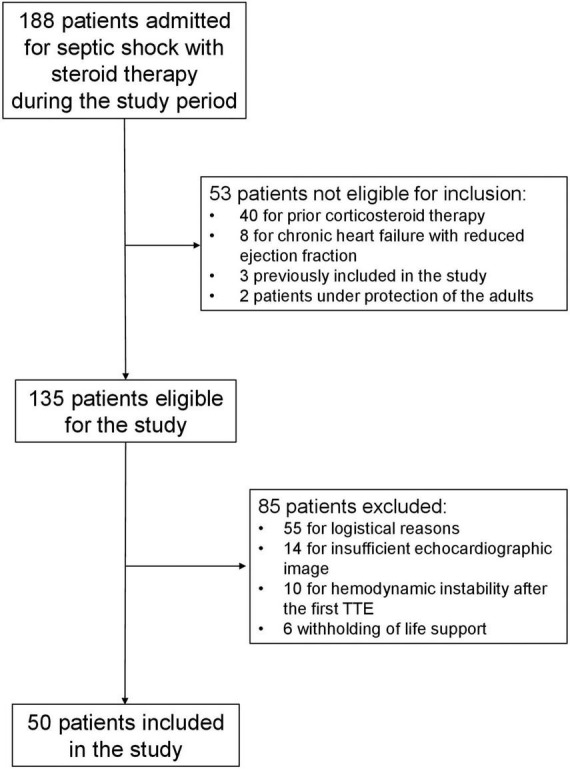
Flow-chart.

During the study period, 188 patients admitted for septic shock and receiving steroid therapy were screened for enrolment. Among these patients, 135 were eligible for the study and 85 were excluded for logistical reasons, poor echogenicity, hemodynamic instability, or withdrawing of life support; finally, 50 patients were included in the study (Figure 1). The logistical reasons for excluding eligible patients from this study were lack of echocardiography-trained operators and urgent examination or procedure. Clinical characteristics, comorbidities and organ failures at inclusion are presented in Table 1. Patients were included a median of 12 [6–20] h after septic shock onset. In this cohort, fludrocortisone was administrated in 41 (82%) patients because the enteral access was not available in all patients (contraindication or waiting for the radiographic control of the gastric tube placement). Echocardiographic and hemodynamic data before and after initiation of low-dose steroids are shown in Table 2. The reproducibility of some echocardiographic variables is reported in Supplementary Table 1. The median delay between the two assessments was 172 (135–218) min. Respiratory settings were comparable between the two hemodynamic assessments.

**TABLE 1:** Clinical characteristics of study patients and organ failures.

Variables	Patients, *n* = 50
Age (years)	65 (55–73)
Male sex	33 (66%)
Body mass index (kg/m^2^)	25.9 (22.2–29.4)
SAPS II at ICU admission	54 (46–84)
***Comorbidities***	
Diabetes mellitus	17 (34%)
Arterial hypertension	22 (44%)
Atrial fibrillation	6 (12%)
Pacemaker implant present	2 (4%)
Ischaemic heart disease	9 (18%)
HFpEF	12 (24%)
Chronic renal replacement therapy	1 (2%)
COPD	3 (6%)
Immunodeficiency	10 (20%)
Cirrhosis	6 (12%)
***Infection source***	
Pulmonary	26 (52%)
Abdominal	5 (10%)
Urinary	6 (12%)
Other origin	13 (26%)
Nosocomial infection	16 (32%)
***Low-dose steroid therapy***	
Hydrocortisone	50 (100%)
Fludrocortisone	41 (82%)
***Organ failures at inclusion***	
Delay between norepinephrine introduction and inclusion (h)	12 (6–20)
SOFA score at inclusion	10 (10–12)
GCS before intubation	15 (9–15)
Sinus rhythm	47 (94%)
Fluid balance since admission (L)	3 (2–4)
Norepinephrine dose (mg/h)	3.8 (2.3–5.5)
Dobutamine use	3 (6%)
Arterial blood lactate (mmol/L)	1.9 (1.3–3.6)
Mechanical ventilation	46 (92%)
Neuromuscular-blocking agent use	22 (44%)
PaO_2_/FiO_2_	198 (153–272)
Tidal Volume (mL)	400 (375–450)
Plateau pressure (cm H_2_O)	21 (18–24)
Positive end expiratory pressure (cm H_2_O)	8 [6–10)
***Outcomes***	
Acute respiratory distress syndrome	23 (46%)
ECMO	4 (8%)
Renal replacement therapy	16 (32%)
Duration of catecholamine (d)	4 (2–6)
Duration mechanical ventilation (d)	9 (6–20)
ICU length of stay (d)	12 (7–22)
Death in ICU	19 (38%)

Values are expressed as number (%) or median (interquartile range). SAPS II, Simplified Acute Physiology Score II; ICU, intensive care unit; HFpEF, heart failure with preserved ejection fraction; COPD, chronic obstructive pulmonary disease; TTE, transthoracic echocardiography; SOFA score, Sequential Organ Failure Assessment; GCS, Glasgow coma scale; PaO_2_, partial pressure of oxygen in arterial blood; FiO_2_, fraction of inspired oxygen; PEEP, positive end-expiratory pressure; ECMO, extracorporeal membrane oxygenation.

**TABLE 2:** Evolution of hemodynamic and echocardiographic parameters in septic shock patients before and after initiation of low-dose steroid therapy.

		Before steroid (*n* = 50)	After steroid(*n* = 50)	*P*-value
***Macrocirculation***				
SAP (mmHg)	*n* = 50	118 (+/-21)	127 (+/-27)	<0.01*
Pulse pressure (mmHg)	*n* = 50	64 (+/-22)	68 (+/-25)	0.049
MAP (mmHg)	*n* = 50	73 (70–78)	79 (71–87)	<0.01*
Heart rate (rpm)	*n* = 50	104 (90–123)	100 (85–112)	0.023*
***Preload and diastolic function***				
LV end-diastolic volume (mL)	*n* = 49	112 (78–139)	107 (83–130)	0.63
E/A ratio at mitral valve	*n* = 44	0.92 (0.77–1.09)	0.84 (0.74–1.14)	0.34
E/e’ ratio at mitral mean valve	*n* = 41	9 (7–14)	9 (7–11)	0.02*
e’ mean (cm. s^–1^)	*n* = 41	9 (6–11)	9 (7–10)	0.16
Respiratory change in VTI LVOT (%)^#^	*n* = 48	5 (0–10)	4 (0–6)	0.08
***LV afterload***				
Ea (mmHg.mL^–1^)	*n* = 50	1.71 (1.37–2.19)	1.90 (1.45–2.27)	0.02*
SVR (mmHg.L^–1^.min)	*n* = 50	893 (740–1,288)	1,065 (746–1,464)	<0.01*
LV end-systolic wall stress (mmHg L)	*n* = 49	5.70 (3.80–7.58)	6.37 (3.93–8.28)	0.46
DAP (mmHg)	*n* = 50	54 (51–57)	58 (53–66)	<0.01*
Diastolic shock index	*n* = 50	1.95 (+/-0.45)	1.75 (+/-0.51)	<0.01*
***LV contractility***				
LVEF (%)	*n* = 49	59 (40–64)	55 (46–63)	0.42
LVEF _*EA*_ (%)	*n* = 49	58 (+/-17)	60 (+/-15)	0.17
LV-GLS (%)	*n* = 47	–13.0 (+/-5.2)	–14.2 (+/-5.1)	0.001*
s’ mean of lateral and septal annulus (cm. s^–1^)	*n* = 42	10.4 (+/-3.8)	11.2 (+/-3.5)	0.02*
s’ at mitral lateral annulus (cm. s^–1^)	*N* = 47	11.3 (+/-4.5)	12.4 (+/-4.5)	0.01*
Ees(sb) (mmHg.mL^–1^)	*n* = 41	1.41 (+/-0.65)	1.64 (+/-0.63)	0.004*
***Global function***				
Stroke volume (mL)	*n* = 50	62 (51–79)	61 (46–81)	0.57
Cardiac Index (L.min^–1^.m^–2^)	*n* = 50	3.5 (2.5–4.3)	3.2 (2.5–4.5)	0.22
Cardiac power index (W.m^–2^)	*n* = 50	0.57 (0.41–0.71)	0.53 (0.39–0.73)	0.34
VAC	*n* = 41	1.30 (0.91–1.79)	1.18 (0.92–1.35)	0.007*

Values are expressed as number (%) or median (interquartile range) or mean (+/-SD), as appropriate. SAP, systolic arterial pressure; MAP, mean arterial pressure; LV, left ventricle; E, early wave of transmitral diastolic blood flow; A, late wave of transmitral diastolic blood flow; E/A, ratio of early to late pulsed-wave Doppler of diastolic transmitral flow velocity; e’, early tissue Doppler diastolic wave velocity at the lateral/septal mitral valve annulus; E/e’, ratio of early pulsed-wave Doppler to early tissue Doppler diastolic wave velocity at the mitral valve annulus; VTI LVOT, velocity-time integral of left ventricular outflow tract; Ea, end-systolic arterial elastance; SVR, systemic vascular resistance; DAP, diastolic arterial pressure; LVEF, left ventricular ejection fraction; LVEF_EA_, afterload-adjusted LVEF (LVEF ×√Ea); LV-GLS, left ventricular global longitudinal strain, S’, tissue Doppler peak systolic wave at mitral annulus; Ees(sb), LV end-systolic maximal elastance by single-beat method; VAC, ventricular-arterial coupling. Paired *t*-test or Wilcoxon paired test were used for Gaussian and non-Gaussian variables, respectively. ^#^Absolute variation index VTI LVOT (%), absolute variation/pre low-dose steroid therapy. *Denote an adjusted *p*-value < 0.05 with Benjamini-Hochberg correction as compared to before with after low-dose steroids therapy.

### Hemodynamics

At inclusion, all patients were receiving norepinephrine, at a median dose of 3.8 (2.3–5.5) mg/h, after a significant fluid resuscitation [median fluid balance of 3 (2–4) L since admission] (Table 1). As compared to baseline values, there was a significant increase in systolic, mean and diastolic arterial pressure after low-dose steroids, along with a significant decrease in heart rate (Table 2).

### Loading conditions

To the exception of LV end-systolic wall stress, all afterload parameters significantly improved after low-dose steroid therapy (with an increase in end-systolic arterial elastance, systemic vascular resistance, diastolic arterial pressure, and a decrease in diastolic shock index) (Table 2). Among preload parameters, only E/e’ significantly decreased after low-dose steroid therapy. However, this variation might not be considered as clinically relevant. We did not find a significant change in global function (stroke volume, cardiac index, and CPI).

### Contractility and ventriculo-arterial coupling

**FIGURE 2:**
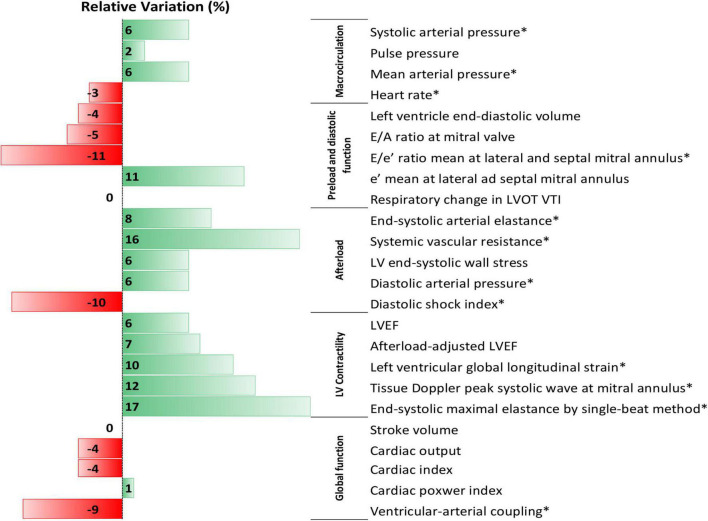
Data bars of median values of percent relative variation in hemodynamic and echocardiographic parameters after initiation of low-dose steroid therapy in septic shock patients. *Denote an adjusted *p*-value < 0.05 with Benjamini-Hochberg correction as compared to before with after low-dose steroids therapy.

To the exception of LVEF, all contractility parameters (LV global longitudinal strain, tissue Doppler peak systolic wave at mitral annulus, and-systolic maximal elastance by single-beat method) significantly improved after low-dose steroid therapy (Table 2, Figure 2, and Supplementary Figure 1). VAC significantly decreased after low-dose steroid therapy; uncoupled VAC was present in 18/41 (44%) before low-dose steroid therapy and 10/41 (24%) after (*p* < 0.01).

### Subgroups analyses

Overall, the hemodynamic and echocardiographic changes induced by low-dose steroids were similar in patients with or without septic myocardial dysfunction (Supplementary Figure 2). MAP responders significantly improved their macrocirculation and afterload parameters, contrary to non-responders (Supplementary Figure 3).

## Discussion

The main findings of this echocardiographic study in septic shock patients were that in addition to their role in peripheral vascular tone (increased afterload), low-dose steroids increased the LV contractility and improved the cardiovascular efficiency.

In this cohort of septic shock patients, the hemodynamic effects of low-dose steroids therapy were coherent with the literature. Indeed, all arterial pressures (systolic, diastolic, and mean) increased in accordance with previous reports on this topic (7). Moreover, all LV afterload parameters assessed (except one) improved after low-doses steroid therapy, also in accordance with previous reports (5, 44). These hemodynamic effects were not homogeneous in the entire cohort as reflected by a greater increase in afterload in MAP responders as compared to non-responders. Although septic myocardial dysfunction was previously shown to be associated with relative adrenal insufficiency (45), we found no difference in the cardiovascular effects of low-dose steroids between patients with and without septic myocardial dysfunction.

In previous studies evaluating hemodynamic effects of hydrocortisone in human septic shock, hydrocortisone increased MAP and systemic vascular resistances, with either decreased cardiac index and heart rate (5, 44) or no obvious effects on cardiac performance (4). The decrease in heart rate may be ascribable to fever control (46). However, these studies did not assess cardiac contractility *per se*. Our finding of increased cardiac contractility with low-dose steroids is in accordance with previous reports suggesting inotropic properties (47) and a possible protective effect on myocardium in the early phase of septic shock (10). Adrenal insufficiency is a cause of cardiac failure (48) and hydrocortisone increased cardiac output in healthy volunteers with hypoaldosteronism (49). The increase in LV contractility induced by low-dose steroids in septic shock was not necessarily expected. Indeed, some LV contractility indices are inversely correlated with afterload during septic shock (9). New echocardiographic tools (tissue Doppler and strain) seem to be more suitable than traditional parameters of systolic function (LVEF) to detect subtle myocardial dysfunction, and to be less influenced by afterload than other LV contractility parameters during sepsis (50). LV strain and especially tissue Doppler s’ were less prone to changes in LV loading conditions (9). Our data showed a significant increase of these parameters of systolic function without any change of LVEF.

End-systolic elastance is a theoretical load independent variable of LV contractile function deduced from the pressure-volume loops, obtained by invasive ventricular catheterization. Herein, we assessed LV end-systolic elastance by the bedside single beat method proposed by Chen et al. (27), which is considered the most accurate non-invasive method and is recommended by guidelines (31). Despite the improvement in afterload with increased end-systolic arterial elastance after initiation of corticosteroid replacement therapy, we detected a significant increase in LV end-systolic elastance by the single-beat method. The reported increase in this variable may be credible since tissue Doppler s’ and strain were also increased. However, Chen’s methods presents the following limitations: (i) it is based on complex assumptions, equations, and regression models; (ii) echo-derived LVEF is incorporated in E_*es(sb)*_ estimate; (ii) the formula of E_*es(sb)*_ uses the ratio of pre-ejection to total systolic time raised to multiple powers, such that small variations in the measurements of time intervals may lead to relatively large changes in the estimated E_*es(sb)*_ value.

VAC reflects the complex interaction between LV performance and arterial load and represents cardiovascular efficiency. When this ratio between arterial elastance and LV elastance at systole is close to 1.0, it indicates an optimal coupling between LV and arterial system resulting in efficient LV stroke work (51). In septic shock, both arterial elastance and LV elastance can profoundly be altered with various degrees of vasoplegia and/or LV myocardial depression. Evaluation of VAC was recently proposed, not only to understand the hemodynamic pathophysiology in septic shock, but also to predict the response to cardiovascular therapies (52). VAC is mathematically related to LVEF. However, this theoretical relationship is correct only if V_*o*_ (an extrapolated value representing the left ventricular volume intercept of the volume axis at a theoretical end-systolic pressure of 0 mmHg) is assumed to be null, like in the normal heart, but this is not the case in heart failure or during hemodynamic instability (53). We herein demonstrate a significant improvement in VAC (tending toward 1.0) after initiation of low-dose steroids with decrease of uncoupled VAC. This result suggest a holistic effect of low-dose steroids on the hemodynamic system, not restricted to the vascular system, but also involving the cardiac contractility with an improved interaction between the heart and the vasculature. However, we cannot exclude a homeometric adaption of the heart. Indeed, according to the Anrep’s effect (54), the heart may adapt to increased loading through an increased contractility to preserve ventricular-arterial coupling.

The main strength of our study is the comprehensive hemodynamic assessment involving modern echocardiographic tools. Steroid replacement therapy improves global outcomes in septic shock patients in some studies but not all. Depicting the effect of steroids on cardiac contractility could be interesting to personalize this therapy. Moreover, cardiac and vascular effect of low-dose steroid therapy may justify extending this therapy to patients in cardiogenic shock (55). Our study also has some limitations. First, the design was monocentric and unblinded, with a relatively limited sample size. Second, the delay between the two echocardiographic evaluations was not constant (time window between 2 and 4 h), due to logistical issues and the pragmatic design. Although this time window is debatable, it was chosen in reference to the previous physiological studies on hemodynamic impact of hydrocortisone and fludrocortisone (49, 56). Third, we did not to assess the hemodynamic effect of low-dose steroids based on adrenal function, and future studies are needed to scrutinize this point. Fourth, excluded patients with unstable hemodynamics may have different cardiac contractile properties and vascular system responses to corticosteroids. Fifth, we could not perform a multivariable because of the limited sample size and the collinearity between some hemodynamic parameters. Sixth, although catecholamine dose and fluid management were kept constant between the two-ultrasound assessments, we cannot exclude their influence on observed hemodynamic modifications. For example, fluids and vasopressors both have inotropic effects *via* Starling and Anrep effect, respectively, among other mechanisms.

Finally, the interpretation of the hemodynamic variations could be limited by the absence of a control group and the open label design, inasmuch as the hemodynamic profile of septic shock may vary with time. Indeed, while the delay between the two-ultrasound assessments was relatively short, the relationship between low-dose steroid therapy and modification of hemodynamics could be influence by the natural course of septic shock.

## Conclusion

We herein report a comprehensive assessment by bedside echocardiography of the hemodynamic impact of low-dose steroids in the early phase of septic shock. We confirmed an increase of arterial pressure, a decrease of heart rate and an improvement of LV afterload after initiation of low-dose steroids. Moreover, we suggested for the first time an increase of LV contractility with improvement of the cardiovascular efficiency as assessed by VAC. However, this possible positive inotropic effect needs to be confirmed in future studies.

## Data availability statement

The raw data supporting the conclusions of this article will be made available by the authors, without undue reservation.

## Ethics statement

The study was approved on July 12, 2018 by the Institutional Ethics Committee, Institutional Review Board (IRB) Mondor (reference number: 2201948) as a component of standard care, and patient consent was waived. Written and oral information about the study was given to the families.

## Author contributions

FB had full access to all of the data in the study and took responsibility for the integrity of the data, and the accuracy of the data analysis. FB and AM designed the study and wrote the manuscript. FB, AC, PM, and KR performed echocardiographies. FB, AC, and PM collected the data. FB, AC, PM, NP, GC, KR, and AM contributed to interpretation of data, drafting of the submitted article, and critical revisions for intellectual content. All authors read and approved the final manuscript.

## Supplementary material

Supplementary Figure 1 Box and whiskers plot of percent relative variation in hemodynamic and echocardiographic parameters after initiation of low-dose steroid therapy in septic shock patients. SAP, systolic arterial pressure; PP, pulse pressure; MAP, mean arterial pressure; HR, heart rate; LVEDV, left ventricle end-diastolic volume; E/A, ratio of early to late pulsed-wave Doppler of diastolic transmitral flow velocity; E/e’, ratio of early pulsed-wave Doppler to early tissue Doppler diastolic wave velocity at the mitral valve annulus; e’, early tissue Doppler diastolic wave velocity at the mitral valve annulus; VTI LVOT, velocity-time integral of left ventricular outflow tract; Ea, end-systolic arterial elastance; SVR, systemic vascular resistance; LV-ESWS, left ventricular end-systolic wall stress; DAP, diastolic arterial pressure; DSI, diastolic shock index; LVEF, left ventricular ejection fraction; LVEF_*EA*_, afterload-adjusted LVEF (LVEF x √Ea); LV-GLS, left ventricular global longitudinal strain; S’, tissue Doppler peak systolic wave at mitral annulus; Ees(sb), left ventricular end-systolic maximal elastance by single-beat method; CO, cardiac output; CI, cardiac index; CPI, cardiac power index; VAC, ventricular arterial coupling.

Supplementary Figure 2 Data bars of median values of percent relative variation in hemodynamic and echocardiographic parameters after initiation of low-dose steroid therapy in septic shock patients, according septic cardiomyopathy. *Denote an adjusted *p*-value < 0.05 with Benjamini-Hochberg correction as compared to septic cardiomyopathy and non-septic cardiomyopathy.

Supplementary Figure 3 Data bars of median values of percent relative variation in hemodynamic and echocrdiographic parameters after initiation of low-dose steroid therapy in septic shock patients, according to steroid responders. *Denote an adjusted *p*-value < 0.05 with Benjamini-Hochberg correction as compared to responders and non-responders.

## Abbreviations

MAP: mean arterial pressure
LV: left ventricle
ICU: intensive care unit
ACCP: American College of Chest Physicians
SCCM: Society of Critical Care Medicine
LVEF: left ventricular ejection fraction
IRB: institutional review board
TTE: left transthoracic echocardiography
PRICES: Preferred Reporting Items for Critical care Echocardiography Studies
E: early wave of transmitral diastolic blood flow
A: late wave of transmitral diastolic blood flow
e’: tissue Doppler diastolic wave velocity at mitral valve annulus
VTI: velocity time integral
LVOT: left ventricle outflow tract
DAP: diastolic arterial pressure
E_*a*_: end-systolic arterial elastance
DSI: diastolic shock index
s’, tissue Doppler imaging: with peak systolic wave
LV-GLS: left ventricle global longitudinal peak systolic strain
E_*es*_: left ventricle end-systolic maximal elastance
E_*es(sb)*_: E_*es*_ estimated by the modified single-beat method
E_*d*_: left ventricular end-diastolic elastance
P_*d*_: diastolic arterial pressure
V_*d*_: left ventricular volume at the onset of ejection
V_0_: volume axis intercept of the end-systolic pressure volume relation
V_*es*_: left ventricular end-systolic volume
SV: stroke volume
[E_*nd(est*)_]: non-invasive estimated normalized left ventricular elastance at the onset of ejection
[E_*nd(avg*)_]: group-average normalized left ventricular elastance at the onset of ejection
VAC: ventricular-arterial coupling
CPI: cardiac power index
LVEF_*EA*_: LVEF adjusted by E_*a*_
SOFA: Sequential Organ Failure Assessment
SAPS: Simplified Acute Physiologic Score
ARDS: of acute respiratory distress syndrome
SD: standard deviation.
