# Supplementary material for: Cardiac and vascular effects of low-dose steroids during the early phase of septic shock: An echocardiographic study
Source: Front Cardiovasc Med. 2022 Sep 26;9:948231. doi: 10.3389/fcvm.2022.948231 (PMC9549363; doi:10.3389/fcvm.2022.948231)
Supplement: Supplementary file 1 [file Table_1.DOCX]

| **TABLE S1** Reproducibility of echocardiography parameters* | |
| --- | --- |
| **Parameters** | **Reproducibility** |
| e’ at lateral mitral annulus, (cm.s^-1^) | 1.25 |
| s’ at mitral lateral annulus, (cm.s^-1^) | 0.93 |
| LV-GLS (%) | 0.87 |
| VTI LVOT (cm) | 1.28 |
| Ees(sb) (mmHg.mL^-1^) | 0.20 |
| Ea (mmHg.mL^-1^) | 0.27 |
| e’: early tissue Doppler diastolic wave velocity, s’: tissue Doppler peak systolic wave at mitral annulus, LV-GLS: left ventricular global longitudinal strain, VTI LVOT: velocity-time integral of left ventricular outflow tract, Ees(sb): LV end-systolic maximal elastance by single-beat method, Ea: end-systolic arterial elastance. *The same sets of recordings were analysed separately by two different ultrasonographers to assess inter-analyser reproducibility, which was expressed as per the British Standards Institution coefficient (twice the standard deviation of the differences in repeated measurements). | |
