# Supplementary figures and images for: Cardiac and vascular effects of low-dose steroids during the early phase of septic shock: An echocardiographic study
Source: Front Cardiovasc Med. 2022 Sep 26;9:948231. doi: 10.3389/fcvm.2022.948231 (PMC9549363; doi:10.3389/fcvm.2022.948231)

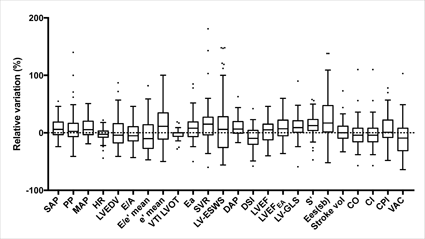

Supplement: Supplementary Figure 1 — Box and whiskers plot of percent relative variation in hemodynamic and echocardiographic parameters after initiation of low-dose steroid therapy in septic shock patients. SAP, systolic arterial pressure; PP, pulse pressure; MAP, mean arterial pressure; HR, heart rate; LVEDV, left ventricle end-diastolic volume; E/A, ratio of early to late pulsed-wave Doppler of diastolic transmitral flow velocity; E/e’, ratio of early pulsed-wave Doppler to early tissue Doppler diastolic wave velocity at the mitral valve annulus; e’, early tissue Doppler diastolic wave velocity at the mitral valve annulus; VTI LVOT, velocity-time integral of left ventricular outflow tract; Ea, end-systolic arterial elastance; SVR, systemic vascular resistance; LV-ESWS, left ventricular end-systolic wall stress; DAP, diastolic arterial pressure; DSI, diastolic shock index; LVEF, left ventricular ejection fraction; LVEFEA, afterload-adjusted LVEF (LVEF x √Ea); LV-GLS, left ventricular global longitudinal strain; S’, tissue Doppler peak systolic wave at mitral annulus; Ees(sb), left ventricular end-systolic maximal elastance by single-beat method; CO, cardiac output; CI, cardiac index; CPI, cardiac power index; VAC, ventricular arterial coupling. [file Image_1.TIFF]

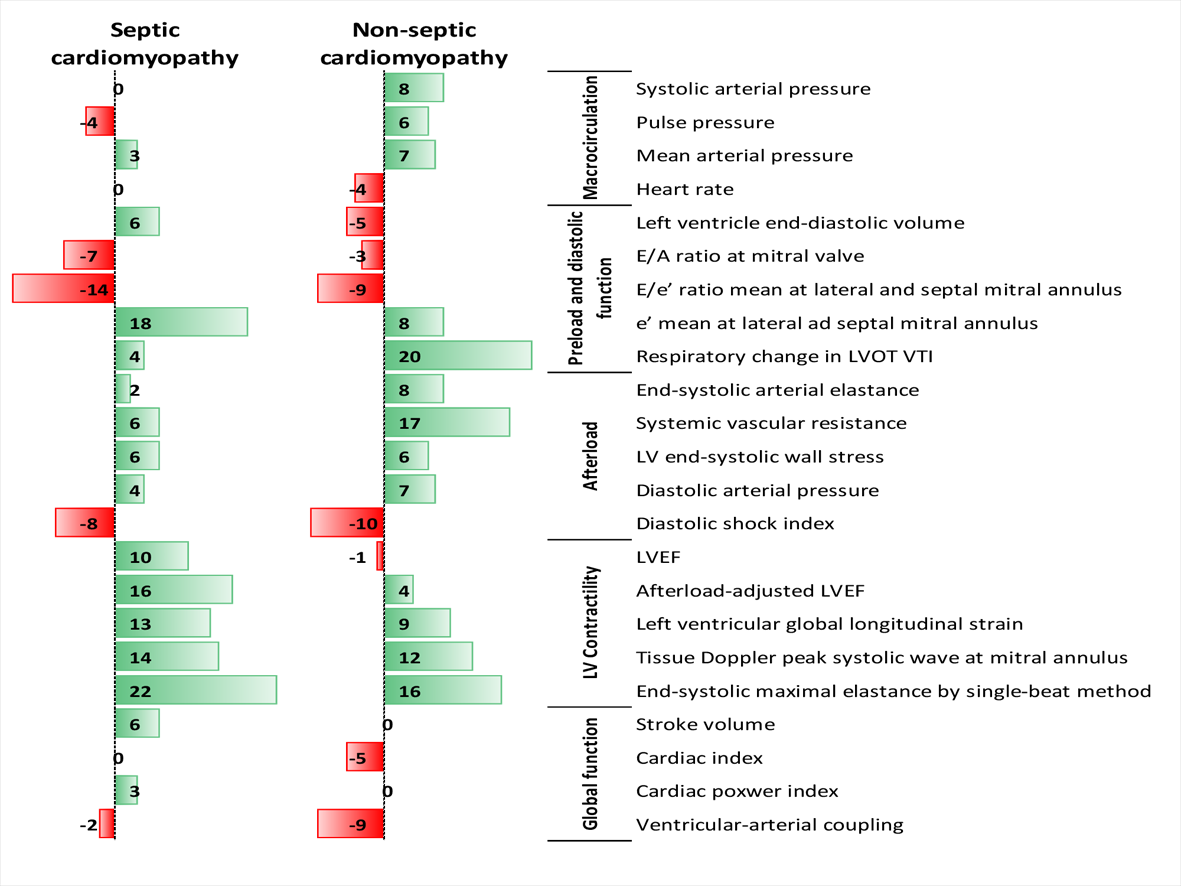

Supplement: Supplementary Figure 2 — Data bars of median values of percent relative variation in hemodynamic and echocardiographic parameters after initiation of low-dose steroid therapy in septic shock patients, according septic cardiomyopathy. *Denote an adjusted p-value < 0.05 with Benjamini-Hochberg correction as compared to septic cardiomyopathy and non-septic cardiomyopathy. [file Image_2.TIFF]

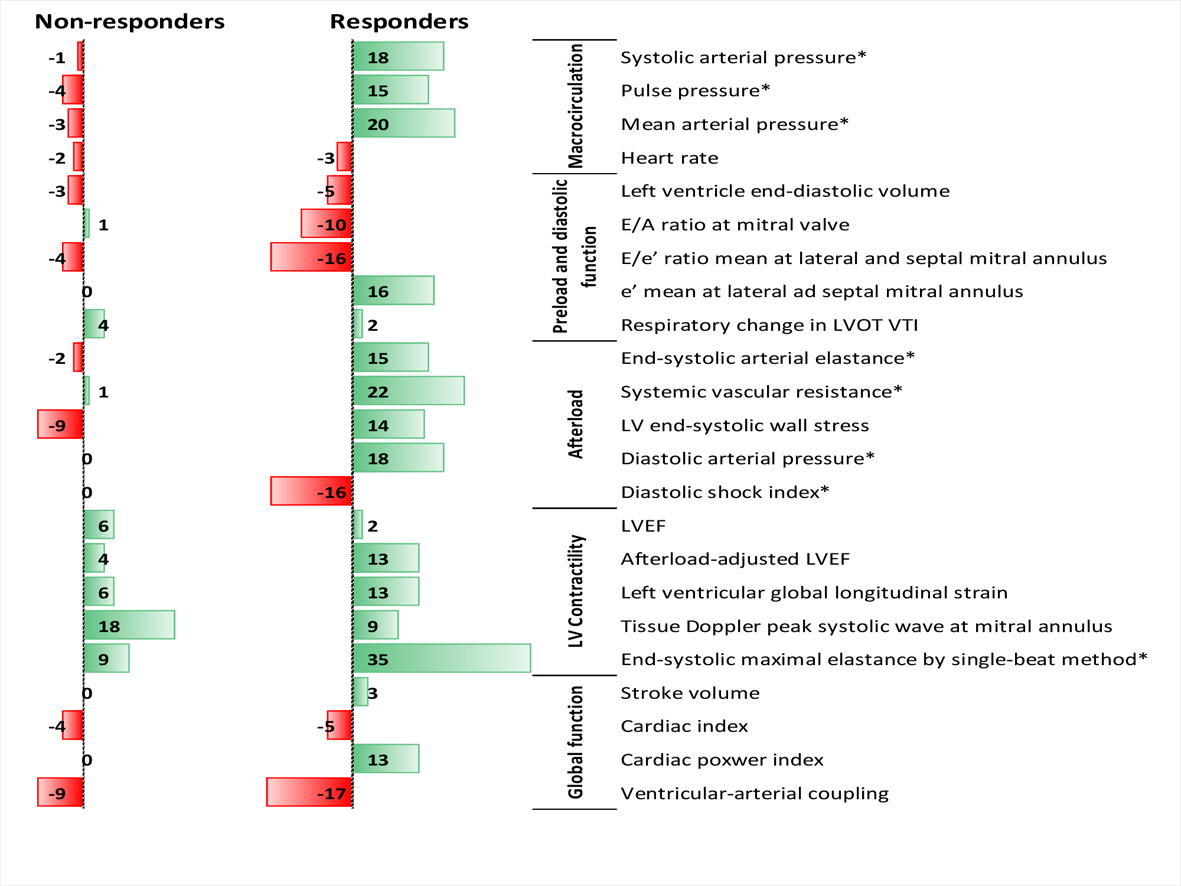

Supplement: Supplementary Figure 3 — Data bars of median values of percent relative variation in hemodynamic and echocrdiographic parameters after initiation of low-dose steroid therapy in septic shock patients, according to steroid responders. *Denote an adjusted p-value < 0.05 with Benjamini-Hochberg correction as compared to responders and non-responders. [file Image_3.TIFF]
